# T_2_STIR preparation for single-shot cardiovascular magnetic resonance myocardial edema imaging

**DOI:** 10.1186/s12968-019-0583-y

**Published:** 2019-11-21

**Authors:** Yanjie Zhu, Dan Yang, Lixian Zou, Yucheng Chen, Xin Liu, Yiu-Cho Chung

**Affiliations:** 1 Paul C. Lauterbur Research Centre for Biomedical Imaging, Shenzhen Institutes of Advanced Technology, Guangdong, 518055 China; 2 Department of Cardiology, West China Hospital, Chengdu, 610041 China; 3 Siemens Healthcare Pte Ltd., 60 MacPherson Road, Singapore, 348615 Singapore

**Keywords:** T_2_STIR, Edema, Single-shot imaging, Short tau inversion recovery

## Abstract

**Background:**

Myocardial edema in acute myocardial infarction (AMI) is commonly imaged using dark-blood short tau inversion recovery turbo spin echo (STIR-TSE) cardiovascular magnetic resonance (CMR). The technique is sensitive to cardiac motion and coil sensitivity variation, leading to myocardial signal nonuniformity and impeding reliable depiction of edematous tissues. T_2_-prepared balanced steady state free precession (T_2_p-bSSFP) imaging has been proposed, but its contrast is low, and averaging is commonly needed. T_2_ mapping is useful but requires a long scan time and breathholding. We propose here a single-shot magnetization prepared sequence that increases the contrast between edema and normal myocardium and apply it to myocardial edema imaging.

**Methods:**

A magnetization preparation module (T_2_STIR) is designed to exploit the simultaneous elevation of T_1_ and T_2_ in edema to improve the depiction of edematous myocardium. The module tips magnetization down to the –z axis after T_2_ preparation. Transverse magnetization is sampled at the fat null point using bSSFP readout and allows for single-shot myocardial edema imaging. The sequence (T_2_STIR-bSSFP) was studied for its contrast behavior using simulation and phantoms. It was then evaluated on 7 healthy subjects and 7 AMI patients by comparing it to T_2_p-bSSFP and T_2_ mapping using the contrast-to-noise ratio (CNR) and the contrast ratio as performance indices.

**Results:**

In simulation and phantom studies, T_2_STIR-bSSFP had improved contrast between edema and normal myocardium compared with the other two edema imaging techniques. In patients, the CNR of T_2_STIR-bSSFP was higher than T_2_p-bSSFP (5.9 ± 2.6 vs. 2.8 ± 2.0*, P* < 0.05) but had no significant difference compared with that of the T_2_ map (T_2_ map: 6.6 ± 3.3 vs. 5.9 ± 2.6, *P* = 0.62). The contrast ratio of T_2_STIR-bSSFP (2.4 ± 0.8) was higher than that of the T_2_ map (1.3 ± 0.1, *P* < 0.01) and T_2_p-bSSFP (1.4 ± 0.5, *P* < 0.05).

**Conclusion:**

T_2_STIR-bSSFP has improved contrast between edematous and normal myocardium compared with commonly used bSSFP-based edema imaging techniques. T_2_STIR-bSSFP also differentiates between fat that was robustly suppressed and fluids around the heart. The technique is useful for single-shot edema imaging in AMI patients.

## Background

As myocardial edema has long T_2_, T_2_-weighted cardiovascular magnetic resonance (CMR) imaging is usually used in the imaging of acute myocardial infarction (AMI) [1–3], where edema commonly occurs. T_2_-weighted turbo spin echo (TSE) combined with short tau inversion recovery (STIR) and dark-blood preparation (STIR-TSE) has been widely used clinically for this purpose [4]. However, the technique is sensitive to cardiac motion, leading to signal loss and hence myocardial signal inhomogeneity. Coil sensitivity variation increases variation of the myocardial signal. Additionally, the stagnant blood at the subendocardial rim would sometimes mimic edematous tissues. These issues of STIR-TSE impede reliable depiction of edematous tissues [5, 6]. Moreover, STIR-TSE is sensitive to arrhythmia and respiratory motion.

Several CMR techniques have been proposed to address these issues of STIR-TSE for myocardial edema imaging. The STIR pulse in STIR-TSE is sometimes replaced by the spectral attenuated inversion recovery (SPAIR) pulse for fat suppression [7] to reduce motion sensitivity. The method is sensitive to main field inhomogeneity. It has also been shown that the technique depends on the proper choice of image readout time [6]. Single-shot T_2_-prepared balanced steady state free precession (T_2_p-bSSFP) [8] shows higher diagnostic accuracy than STIR-TSE for edema imaging [9, 10]. It is robust to cardiac motion and arrhythmia and avoids the bright subendocardial rims caused by stagnant blood that mimic myocardial edema as in STIR-TSE. However, it is sensitive to coil sensitivity variation, and multiple acquisitions are commonly needed to improve image SNR. Another method, ACUT_2_E [11], improves T_2_ weighting intrinsic in bSSFP by using 180° excitation pulses. Yet, high flip angles for the excitation pulses might not be achieved due to the specific absorption rate (SAR) limit and the technique’s sensitivity to transmit (B_1_+) field inhomogeneity, especially at a high field. Similar to STIR-TSE, ACUT_2_E is a segmented sequence and is sensitive to arrhythmia and respiratory motion as well.

T_2_ mapping [12–15] CMR was proposed for myocardial edema imaging [16]. T_2_ quantification can help objectively distinguish between normal and edematous myocardium. It can also detect myocarditis with systematic T_2_ elevation in myocardium. In this method, several T_2_p-bSSFP images with different echo times are acquired over multiple heart beats. The T_2_ map is then obtained through pixelwise curve fitting of these T_2_-weighted images upon image registration. The technique is sensitive to arrhythmia and requires breathholding. Moreover, the accuracy of T_2_ maps depends on multiple factors, e.g., heart rate, fitting model, the patient’s breathholding ability, off-resonance effect, etc. [13, 17–19].

Recent parametric mapping studies on myocardial edema reaffirmed earlier findings [20] that both T_1_ and T_2_ values are increased in edematous tissues [21, 22]. Although the native T_1_ of myocardium also increases in the presence of fibrosis, there is no measurable fibrosis expected at 2 to 5 days post-reperfusion. Therefore, any increase in myocardial T_1_ most likely comes from edema. Thus, a CMR sequence that exploits the elevation of both T_1_ and T_2_ values may yield an edema detection method at a higher specificity. bSSFP cine, whose signal is proportional to √(T_2_/T_1_), has been proposed to depict edematous tissue [23]. However, the signal equation shows that the signal increase due to T_2_ elevation is attenuated by the concomitant increase in T_1_. The resulting signal change is small. The method is therefore sensitive to coil sensitivity variation. Postcontrast bSSFP cine shortens myocardial T_1_ values and improves the contrast between edematous and normal myocardium [24]. The use of contrast agents increases patient risk. Also, the image contrast depends on contrast dosage and the delay time after contrast injection.

This study proposes a novel single-shot CMR technique for edema imaging. The new technique uses a magnetization preparation module called T_2_STIR (T_2_ prepared inversion with STIR) to exploit the simultaneous elevation of T_1_ and T_2_ in edematous tissues for improved differentiation of edematous and normal myocardium while suppressing fat. The module is combined with single-shot bSSFP, making it insensitive to arrhythmia and breathing motion. The performance of the resulting single-shot sequence is assessed by simulation, a phantom study, and in vivo studies in healthy subjects. The technique is finally applied to a small number of patients with known myocardial edema to demonstrate its clinical feasibility. The preliminary work of this study was first reported in [25].

## Theory

### The T_2_STIR preparation module

**Fig. 1:**
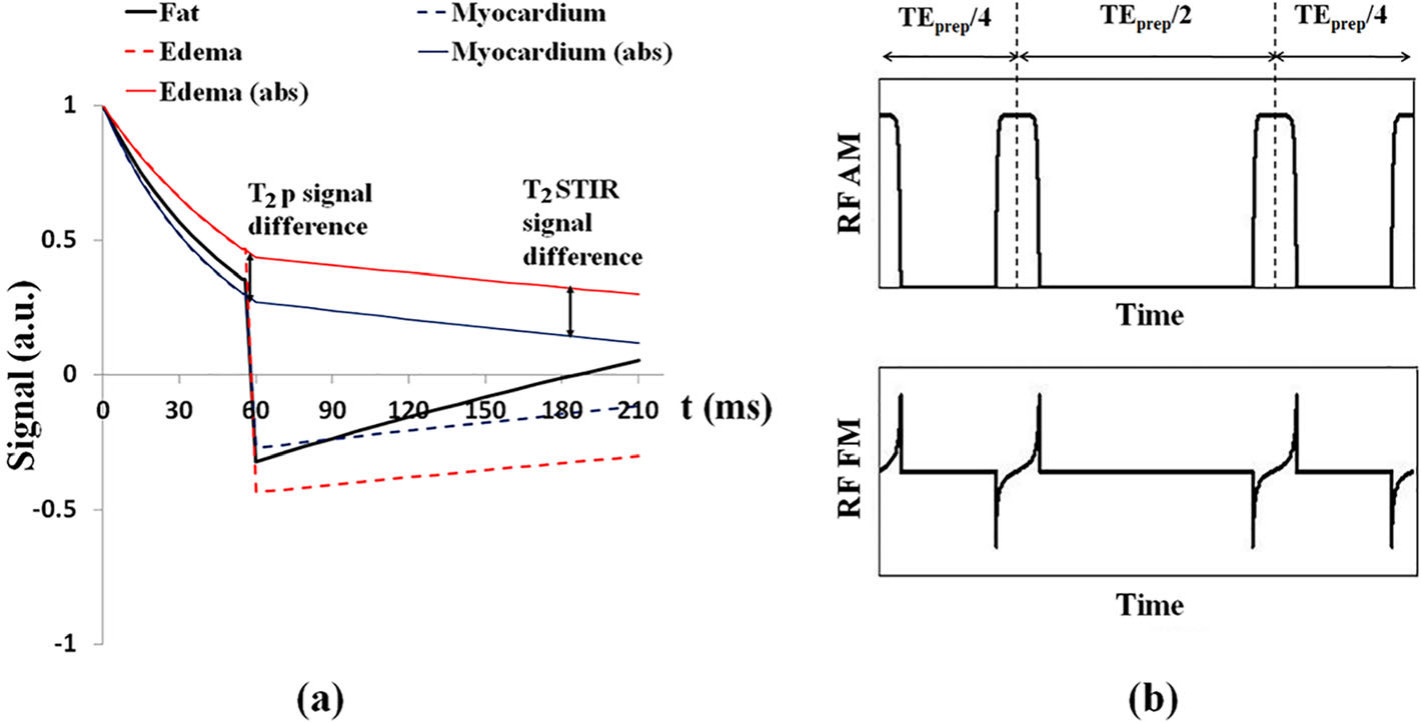
**a** The difference between a T_2_ preparation module and the T_2_ short tau inversion recovery (STIR) module in preparing the magnetization of the myocardial tissues for imaging. The T_2_STIR module tips the magnetization down to the –z axis after T_2_ preparation. **b** The amplitude and frequency modulation of the adiabatic T_2_STIR magnetization preparation module

Figure 1 (a) shows the signal evolutions of edema and normal myocardium with the T_2_STIR module. Contrast of the T_2_STIR module between edema and normal myocardium is generated by T_2_ preparation together with spin inversion. Upon inversion of the T_2_ prepared magnetization, the longitudinal magnetization of a tissue is given by:

1 $$ {\mathrm{M}}_{\mathrm{z}}=-{\mathrm{M}}_0\bullet {\mathrm{e}}^{-{\mathrm{T}\mathrm{E}}_{\mathrm{prep}}/{\mathrm{T}}_2} $$

Here, TE_prep_ is the echo time of the T_2_ preparation module, and T_2_ is the spin-spin relaxation time of the tissue. The resulting magnetization is then sampled at TI_fat_, the fat null point, which is given by:

2 $$ {\mathrm{T}\mathrm{I}}_{\mathrm{fat}}={\mathrm{T}}_{1,\mathrm{fat}}\bullet \ln \left(1+{\mathrm{e}}^{-{\mathrm{T}\mathrm{E}}_{\mathrm{prep}}/{\mathrm{T}}_{2,\mathrm{fat}}}\right) $$

where T_1, fat_ and T_2, fat_ are the T_1_ and T_2_ relaxation times for fat.

After spin inversion, normal myocardium recovers faster than edematous myocardium (see Fig. 1(a)). With magnitude reconstruction, inverted magnetization is “reverted”: edematous tissues now have higher signal intensity than normal myocardium. The inversion and the partial recovery over TI accentuate the signal difference between edema and normal myocardium generated by T_2_ preparation alone. Here, the choice of TI to null fat is similar to the STIR pulse. This new magnetization preparation module is hereby referred to as T_2_STIR.

Equation (2) shows that TI_fat_ is determined by T_1_ and T_2_ of fat (which are 449 ms and 53 ms at 3 T, respectively [26]) and the TE_prep_ used in the T_2_ preparation part of the pulse. For instance, at TE_prep_ = 60 ms, TI_fat_ would be 125 ms.

### T_2_STIR-prepared single-shot edema imaging

Figure 1(b) shows the implementation of the T_2_STIR module designed based on the modified B_1_-insensitive rotation pulse (mBIR-4) [27]. It has three components: a reverse adiabatic half passage (rAHP) pulse, followed by an adiabatic full passage (AFP) pulse and finally an adiabatic half passage (AHP) pulse. The AFP pulse is used twice to reduce the module’s sensitivity to B_0_ and B_1_+ field inhomogeneity [28]. The phase shift and delay times among the four pulses are designed to flip the transverse magnetization down to the –M_z_ direction after T_2_ preparation. The rAHP pulse, AFP pulse and AHP pulse are 2.56 ms, 5.12 ms and 2.56 ms long, respectively, giving a minimum TE_prep_ of 15.36 ms. The frequency sweep is 4.88 kHz. A spoiler gradient at the end of the module destroys any coherent transverse magnetization after the preparation module. Figure 1(b) shows the amplitude and frequency modulations of this adiabatic T_2_STIR preparation pulse based on the tan/tanh modulation function.

Imaging is performed using the single-shot bSSFP readout, similar to that of T_2_p-bSSFP. Linear flip angle (LFA) pulses for catalysis [29] and linear reordering are used to minimize transient oscillations in bSSFP at the start of acquisition [12]. The acquisition is timed to mid-diastole. The sequence is hereafter referred to as T_2_STIR-bSSFP.

In T_2_STIR-bSSFP, TI_fat_ is the time from the end of the T_2_STIR module to the k-space center of the bSSFP readout. Let TI_fill_ be the fill time between the end of the T_2_STIR module and the start of bSSFP readout. Then,

3 $$ {\mathrm{TI}}_{\mathrm{fill}}={\mathrm{TI}}_{\mathrm{fat}}-\left(\mathrm{R}+\mathrm{N}\right)\bullet \mathrm{TR} $$

where R is the number of LFA pulses, and N is the number of lines acquired between the end of the LFA pulse train and the k-space centerline. As TI_fill_ ≥ 0, the choice of R and N together set the lower limit of TI_fat_, and hence the maximum TE_prep_ allowed. Experience from T_2_p-bSSFP shows that a TE_prep_ of 60 ms is commonly used to generate T_2_-induced signal differences for the differentiation of edema and normal myocardium [8]. If this same TE_prep_ is used in the T_2_STIR module, it corresponds to a TI_fat_ of approximately 125 ms and would satisfy Eq. (3).

## Methods

### Simulation

T_2_STIR-bSSFP was compared with T_2_p-bSSFP with TE_prep_ varying from 40 ms to 70 ms through Bloch equation simulation using MATLAB (version 2017a, The MathWorks Inc., Natick, Massachusetts, USA). The parameters for bSSFP readout used in the simulation were as follows: 10 LFA pulses, 22 phase encoding lines before the k-space center, flip angle = 60°, TE/TR = 1.3/2.6 ms. The T_1_ and T_2_ values were 1139 ms and 52 ms for normal myocardium and 1434 ms and 75 ms for edematous tissue. They were the relaxation parameters of the compartments in the phantom study below.

### Phantom study

A phantom experiment was performed to evaluate the performance of T_2_STIR-bSSFP and compare it with T_2_p-bSSFP and T_2_ mapping [12]. The sequence was implemented on a 3 T clinical CMR system (TIM TRIO, Siemens Healthineers, Erlangen, Germany) for all experiments. A two-compartment phantom, one for normal myocardium and the other for edema, was built with agar doped with NiCl_2_. The T_1_ values of the compartments were measured using an inversion recovery spin echo sequence (TR = 10 s, TI changing from 100 ms to 3600 ms). The T_2_ values were measured using a spin echo sequence (TR = 10 s, TE varying from 10 ms to 150 ms). The T_1_ and T_2_ values were 1139 ms and 52 ms for normal myocardium compartment and 1434 ms and 75 ms for edema compartment, respectively.

The phantom was imaged with T_2_STIR-bSSFP, T_2_p-bSSFP, and T_2_ mapping [12]. Simulated electrocardiogram (ECG) with an RR interval of 800 ms was used. The value of TE_prep_ in the T_2_STIR and T_2_p modules was varied from 40 ms to 70 ms in 5 ms increments. Imaging parameters identical to all sequences were as follows: FOV = 240 × 83 mm^2^, matrix size = 128 × 44, pixel size = 1.9 × 1.9 mm^2^, slice thickness = 8 mm, TE/TR = 1.3/2.6 ms, bandwidth = 1447 Hz/pixel, flip angle = 60°. Parallel imaging was not used, allowing accurate estimation of signal and noise in the images. The experiments were repeated four times. Images were then averaged for analysis.

### Healthy subject study

A protocol for in vivo imaging was developed and evaluated in healthy subjects. Blood was used as a surrogate for edema because its T_1_ and T_2_ values were both longer than normal myocardium. The study was approved by the institutional review board (IRB) at Shenzhen Institutes of Advanced Technology. Seven healthy subjects (5 males, 26 ± 3 years) were recruited, and informed consent was obtained. For each subject, three short axis slices (basal, mid-ventricular and apical) and one horizontal long axis of the heart were acquired using T_2_STIR-bSSFP and T_2_p-bSSFP. Imaging parameters for both single-shot sequences were identical: FOV = 360 × 260 mm^2^, matrix size = 192 × 138, pixel size = 1.9 × 1.9 mm^2^, slice thickness = 8 mm, TE/TR = 1.3/2.6 ms, flip angle = 60°, bandwidth = 1447 Hz/pixel, phase resolution = 75%, TE_prep_ = 60 ms, and TI_fill_ was 20 ms (TI_fat_ = 125 ms) in T_2_STIR-bSSFP. GRAPPA rate 2 with 24 auto-calibration lines was used to reduce the image acquisition window.

### Patient study

The ability of the three imaging techniques (T_2_STIR-bSSFP, T_2_p-bSSFP and T_2_ mapping) in detecting edema was evaluated in AMI patients. The study was approved by the IRB of West China Hospital, where patient recruitment and scanning were carried out. Seven patients (5 males, 60 ± 10 years) with ST-segment elevation myocardial infarction (STEMI), identified by clinical presentation, ECG and coronary angiograms, were recruited after reperfusion. Informed consent was obtained from each patient. The patients were examined on days 2 to 5 post-reperfusion using the institution’s standard CMR protocols, including STIR-TSE, T_1_ maps [30] and T_2_ maps [12] on a 3 T CMR scanner (TIM TRIO, Siemens Healthineers). The patients were then imaged with T_2_STIR-bSSFP and T_2_p-bSSFP (using the protocols in the healthy subject study). Late gadolinium enhancement (LGE) images were then acquired for each patient approximately 10 min after the injection of gadolinium-based contrast agent (0.15 mmol/kg, Magnevist, Bayer, Whippany, New Jersey, USA). Imaging parameters of the relevant sequences are listed in Table 1.

**Table 1:** Imaging parameters used in the patient study

	T_1_ mapping	T_2_ mapping	T_2_p/T_2_STIR-bSSFP	STIR-TSE^a^	LGE
TE/TR	1.15/2.7 ms	1.09/2.5 ms	1.3/2.6 ms	62/2 RR	3.4/6.6 ms
Image matrix	256 × 216	192 × 160	192 × 144	256 × 216	256 × 216
Resolution	1.4 × 1.9 mm	1.9 × 2.5 mm	1.9 × 2.5 mm	1.4 × 1.4 mm	1.4 × 1.4 mm
Readout	bSSFP	bSSFP	bSSFP	TSE	GRE
Flip angle	35^o^	35 ^o^	60 ^o^	90^o^	20^o^
Bandwidth (Hz/pixel)	1085	1447	1447	781	287
Echo train	n.a.^b^	n.a.	n.a.	15	n.a.
TE_prep_	n.a.	0, 25, 55 ms	60 ms	n.a.	n.a.
Scan time (heartbeats)	11	7	1	12	8
Acquisition	“Single-shot”, multiple acq.		Single-shot	Segmented	Segmented
Parallel imaging	GRAPPA rate 2				

^a^The STIR-TSE sequence was not included in the sequence performance comparison LGE, late gadolinium enhancement  TE, echo time  TR, repetition time

^b^ n.a. means “not applicable”

### Data analysis

The performance of T_2_STIR-bSSFP was assessed by (1) the signal difference in the simulation or the contrast-to-noise ratio (CNR) in images and (2) the contrast ratio between edema and normal myocardium [20]. In the images, signal intensity (SI) was given by the mean signal of the region of interest (ROI). Noise was given by the standard deviation (SD) of normal myocardium [31]. The equations to find the signal difference, signal to noise ratio (SNR), CNR, and contrast ratio for T_2_p-bSSFP and T_2_STIR-bSSFP, as well as the calculation of SNR, CNR and contrast ratio for the T_2_ maps, are defined in the Additional file 1.

In the phantom study, ROIs were manually drawn for each compartment. The signal difference and the contrast ratio between the normal and edematous myocardium compartments of the phantom for T_2_STIR-bSSFP and T_2_p-bSSFP at each TE_prep_ were found. The correlation between the simulation and the phantom study was assessed by linear regression. The contrast ratio of the T_2_ value between the normal and edematous myocardium compartments in the T_2_ map was also calculated.

For healthy subjects, the ROIs of myocardium and blood pool were manually drawn for each subject. The myocardial signal was measured at the septum. Blood signals were measured at the center of the blood pool in the left ventricle. The CNRs and contrast ratios between the myocardium and the blood (surrogate for edema) of T_2_STIR-bSSFP and T_2_p-bSSFP were calculated for each slice. The average CNRs and contrast ratios over all the slices were recorded for each sequence.

For AMI patients, the ROI of remote myocardium was manually drawn on the LGE images by an experienced reader (5-year CMR experience). This ROI was copied and pasted on T_1_ map and T_2_ map to keep the ROI position the same as on the LGE image. The ROIs on T_1_ and T_2_ maps were slightly adjusted to ensure the entire ROI was inside the myocardium if the myocardium on T_1_ or T_2_ map was not aligned with the myocardium on LGE image due to motion. The mean and SD values of these regions were calculated for the T_1_ and T_2_ maps. Myocardial regions with T_1_ and T_2_ values larger than the mean + 2 SD of the remote myocardium were identified as edematous regions. At the regions where both T_1_ and T_2_ were elevated (the two parametric maps may have slightly different shapes), contours for signal measurement were manually drawn inside these regions. Regions of microvascular obstructions (identified on LGE images) and artifacts (i.e., motion artifact, banding artifact), if present, were excluded from the ROIs. The CNR and contrast ratio between the myocardium and edema in each image were calculated for T_2_STIR-bSSFP, T_2_p-bSSFP, and T_2_ mapping. The two performance indices were then averaged among the patients for each imaging method compared in this study. The CNR and contrast ratio differences (given as the mean ± SD) among the three sequences compared were tested for statistical significance. The data were tested for a normal distribution using the Kolmogorov-Smirnov Test. The CNR and the contrast ratio among the different methods were compared using the Mann-Whitney U-test. Statistical significance was set at *P* < 0.05. The extents of edema determined by the different methods are shown in Additional file 1. Note that the STIR-TSE was not included in the sequence comparison in this study since previous clinical studies already showed that T_2_p-bSSFP has better diagnostic accuracy than STIR-TSE [8, 10].

## Results

### Simulation

**Fig. 2:**
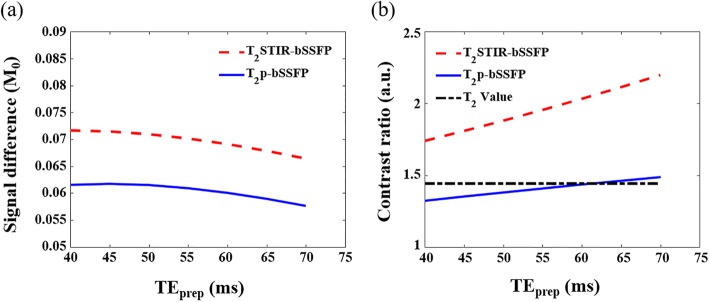
The change in (**a**) signal differences and (**b**) contrast ratios between edematous and normal myocardium with TE_prep_ in T_2_STIR-balanced steady state free precession (bSSFP) and T_2_p-bSSFP in simulation. The signal differences are defined in normalized units of equilibrium magnetization (M_0_). The contrast ratio of T_2_ value between edematous and normal myocardium is also shown for comparison. T_2_STIR-bSSFP has a higher signal difference than T_2_p-bSSFP and has the highest contrast ratio among the three edema imaging sequences. The contrast ratios from T_2_p-bSSFP and the T_2_ map are similar, as both were T_2_-prepared in the same way. The contrast ratio of T_2_STIR-bSSFP increases faster with TE_prep_ than that of T_2_p-bSSFP

The variation in signal difference and contrast ratio between normal myocardium and edema with TE_prep_ in T_2_STIR-bSSFP and T_2_p-bSSFP are shown in Fig. 2. The contrast ratio of the T_2_ values between the two compartments is also shown in Fig. 2(b) for comparison. The signal differences in T_2_STIR-bSSFP are higher than those of T_2_p-bSSFP and are insensitive to TE_prep_ in both cases. Meanwhile, the contrast ratios of the two sequences increase with TE_prep_. The contrast ratio increases faster with TE_prep_ for T_2_STIR-bSSFP than for T_2_p-bSSFP. T_2_STIR-bSSFP has the highest contrast ratio among the three edema imaging sequences compared. Note that the contrast ratios from T_2_p-bSSFP and the T_2_ map are similar, as both were T_2_ prepared in the same way.

At TE_prep_ = 60 ms, the signal difference between edema and normal myocardium in T_2_STIR-bSSFP is 15% higher than that of T_2_p-bSSFP. At the same TE_prep_, the contrast ratios between edema and normal myocardium for T_2_p-bSSFP and T_2_ map are approximately the same and are approximately 30% lower than that of T_2_STIR-bSSFP.

### Phantom study

**Fig. 3:**
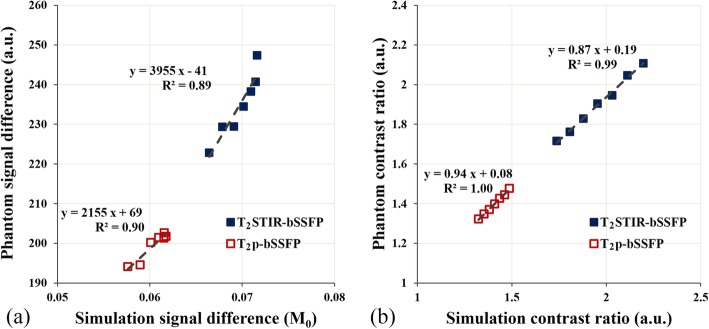
**a** Correlation between the signal differences in the simulation and the phantom study for T_2_p-bSSFP and T_2_STIR-bSSFP. **b** Correlations between the contrast ratios of the simulation and the phantom study for T_2_p-bSSFP and T_2_STIR-bSSFP

The signal differences of the simulation and the phantom study showed strong correlations for T_2_p-bSSFP (*R*^*2*^ = 0.90) and T_2_STIR-bSSFP (*R*^*2*^ = 0.89) (Fig. 3(a)). Excellent correlations between the contrast ratios of the simulation and the phantom study were obtained for T_2_p-bSSFP (*R*^*2*^ = 0.99, slop = 0.99) and T_2_STIR-bSSFP (*R*^*2*^ = 0.98, slop = 0.98) (Fig. 3(b)). The T_2_ values measured by T_2_ mapping were 74 ms and 55 ms for the two phantom compartments representing edema and normal myocardium, respectively. The corresponding contrast ratio for the T_2_ map (1.36) is very close to the expected value (1.44).

### In vivo experiments

#### Healthy subject study

The average CNR between myocardium and blood (surrogate for edema) of T_2_STIR-bSSFP was higher than that for T_2_p-bSSFP (29.6 ± 8.3 vs. 19.0 ± 5.7, *P* < 0.001). The average contrast ratio of T_2_STIR-bSSFP was also higher than that of T_2_p-bSSFP (6.1 ± 0.9 vs. 2.9 ± 0.5, *P* < 0.001).

#### Patient study

**Fig. 4:**
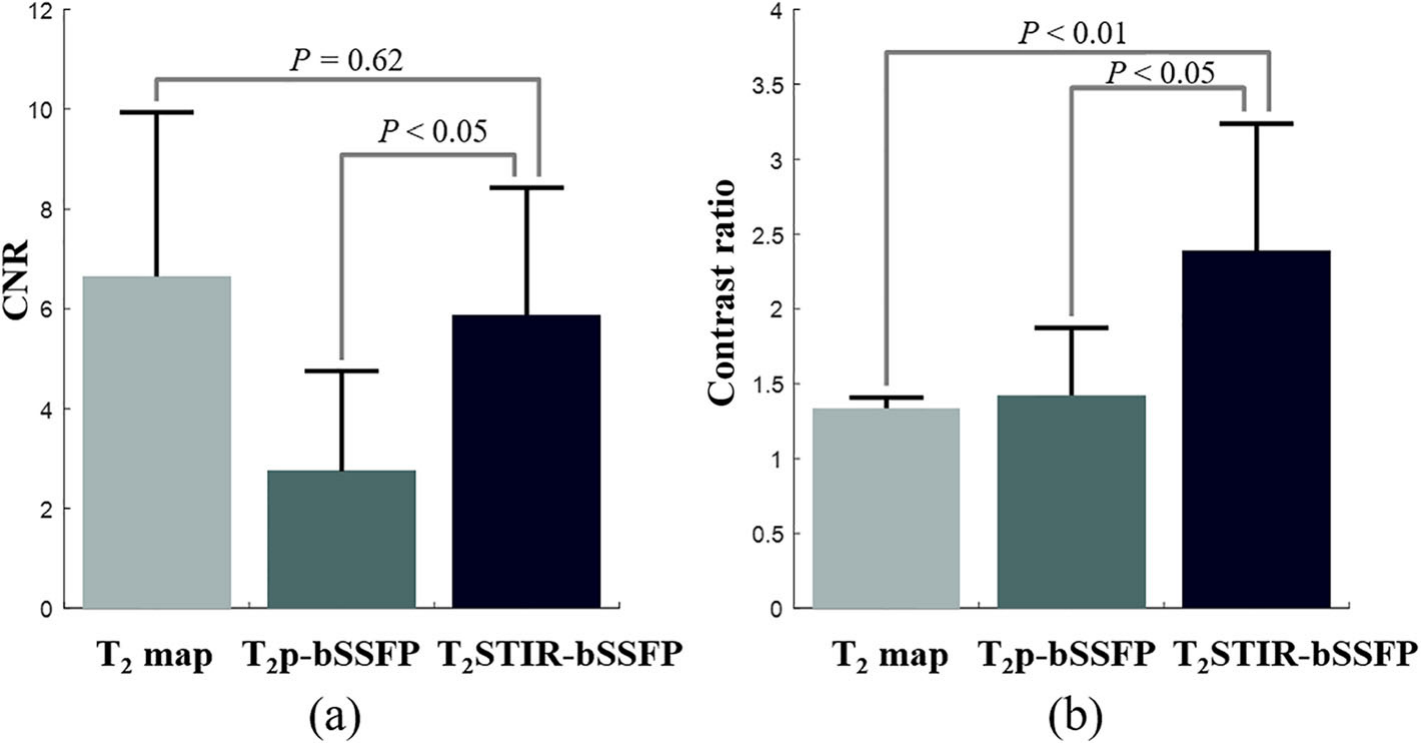
Comparison of (**a**) contrast to noise ratio (CNR)s and (**b**) contrast ratios among T_2_ map, T_2_p-bSSFP, and T_2_STIR-bSSFP. The CNR of T_2_STIR-bSSFP was higher than that of T_2_p-bSSFP, and the contrast ratio of T_2_STIR-bSSFP was higher than that of T_2_ map and T_2_p-bSSFP

Among the seven AMI patients, four had microvascular obstruction. They were identified and excluded from the corresponding ROIs. Table 2 summarizes the mean and SD of SI and SNR for normal and edematous myocardium for all the edema imaging techniques compared. Among the three methods, T_2_ map has the smallest coefficient of variation (which is the reciprocal of SNR). Figure 4 shows the CNRs and the contrast ratios of T_2_ map, T_2_p-bSSFP, and T_2_STIR-bSSFP in bar graphs. The CNR of T_2_STIR-bSSFP was higher than that of T_2_p-bSSFP (5.9 ± 2.6 vs. 2.8 ± 2.0*, P* < 0.05). There was no significant difference between the CNRs of the T_2_ map and T_2_STIR-bSSFP (T_2_ map: 6.6 ± 3.3 vs. 5.9 ± 2.6, *P* = 0.62). However, the contrast ratio of T_2_STIR-bSSFP (2.4 ± 0.8) was higher than that of the T_2_ map (1.3 ± 0.1, *P* < 0.01) and T_2_p-bSSFP (1.4 ± 0.5, *P* < 0.05).

**Table 2:** Summary of average mean and standard deviation of the signal and the signal to noise ratio (SNR) for normal and edematous myocardium

	T_1_ map	T_2_ map	T_2_p-bSSFP	T_2_STIR-bSSFP
Myocardial signal	1218 ± 76 ms	43 ± 3 ms	90 ± 11	37 ± 9
Edema	1493 ± 48 ms	57 ± 4 ms	124 ± 16	86 ± 14
SNR of normal myocardium	34.7 ± 8.2	16.3 ± 5.4	9.9 ± 4.2	5.0 ± 1.5
SNR of edema	41.7 ± 10.7	21.9 ± 8.1	12.8 ± 4.8	9.9 ± 3.1

**Fig. 5:**
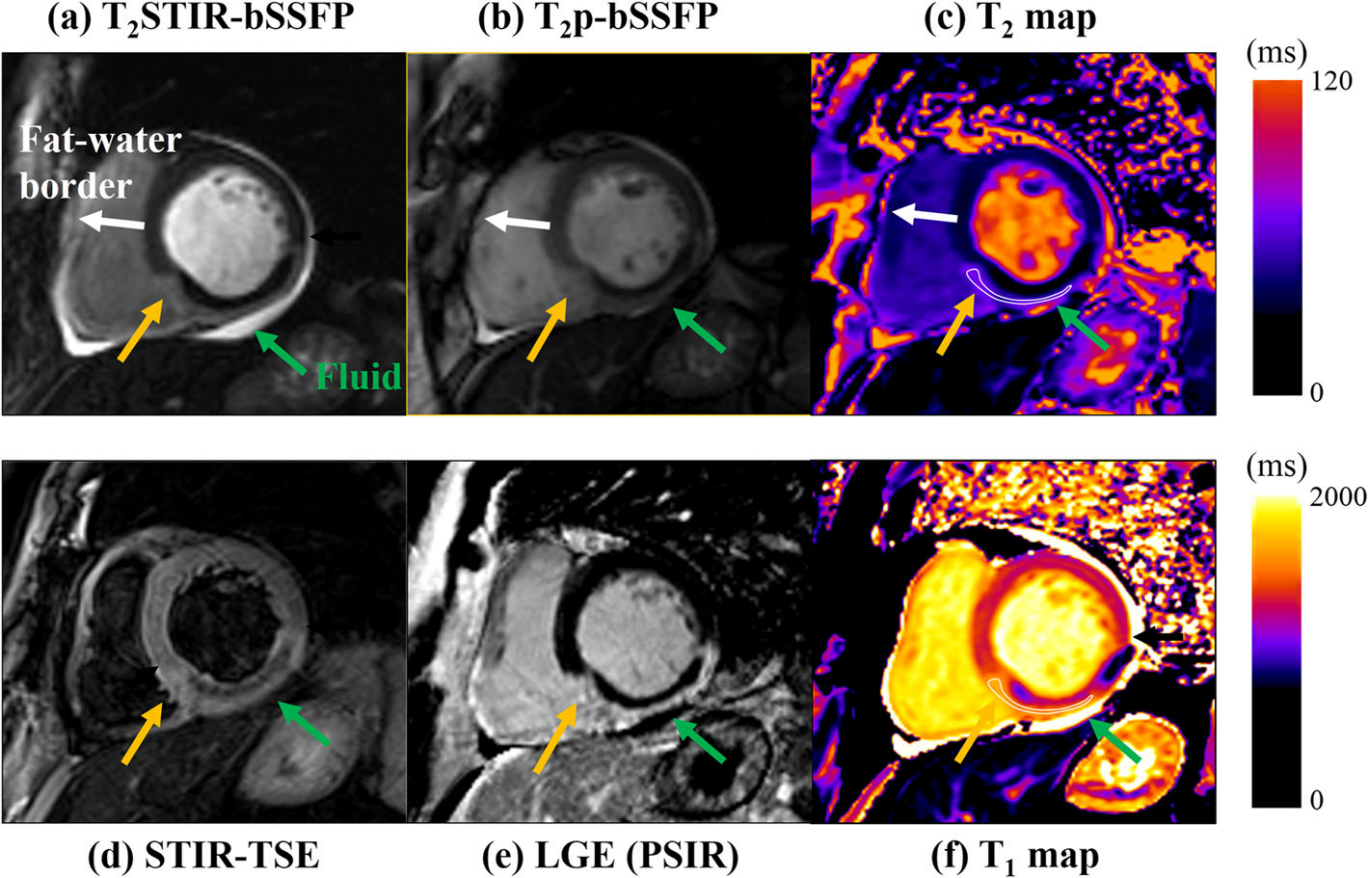
Images from one AMI patient. The location of elevated T_1_ and T_2_ in the two parametric maps (Fig. 5(**c**) and (**f**)) corresponded to edema. The dark core inside the edema/infarct in the late gadolinium enhancement (LGE, Fig. 5(**e**)) most likely corresponded to microvascular obstruction, which had both low T_1_ and T_2_ in the parametric maps. The position of the dark core within the edema/infarct in LGE matched well with the STIR-TSE (Fig. 5(**d**)), T_2_STIR-bSSFP (Fig. 5(**a**)) and T_2_p-bSSFP (Fig. 5(**b**)) images. In the fat-suppressed T_2_STIR-bSSFP image, the tissue indicated by the yellow arrow was likely pericardial fluid (yellow arrow). In the T_2_p-bSSFP image, both epicardial fat and pericardial fluid were bright. The images of T_2_STIR-bSSFP and T_2_p-bSSFP were windowed to the same level to facilitate visual comparison

Figure 5 shows the images from one AMI patient. The infarct region in the LGE image matched the location where both T_1_ and T_2_ were elevated in the two parametric maps. The dark core inside the edema/infarct in LGE (where T_1_ and T_2_ in the parametric maps were both low) most likely corresponded to microvascular obstruction. The signal from the normal myocardium in T_2_STIR-bSSFP was lower than that of T_2_p-bSSFP, making the edematous region in T_2_STIR-bSSFP obvious. Note the bright signal surrounding the heart (yellow arrow) in both single-shot bSSFP images. The fat-suppressed T_2_STIR-bSSFP image suggested that it was likely pericardial fluid. In the T_2_p-bSSFP image, both epicardial fat and pericardial fluid were bright. Edema was also depicted in STIR-TSE (Fig. 5(d)).

**Fig. 6:**
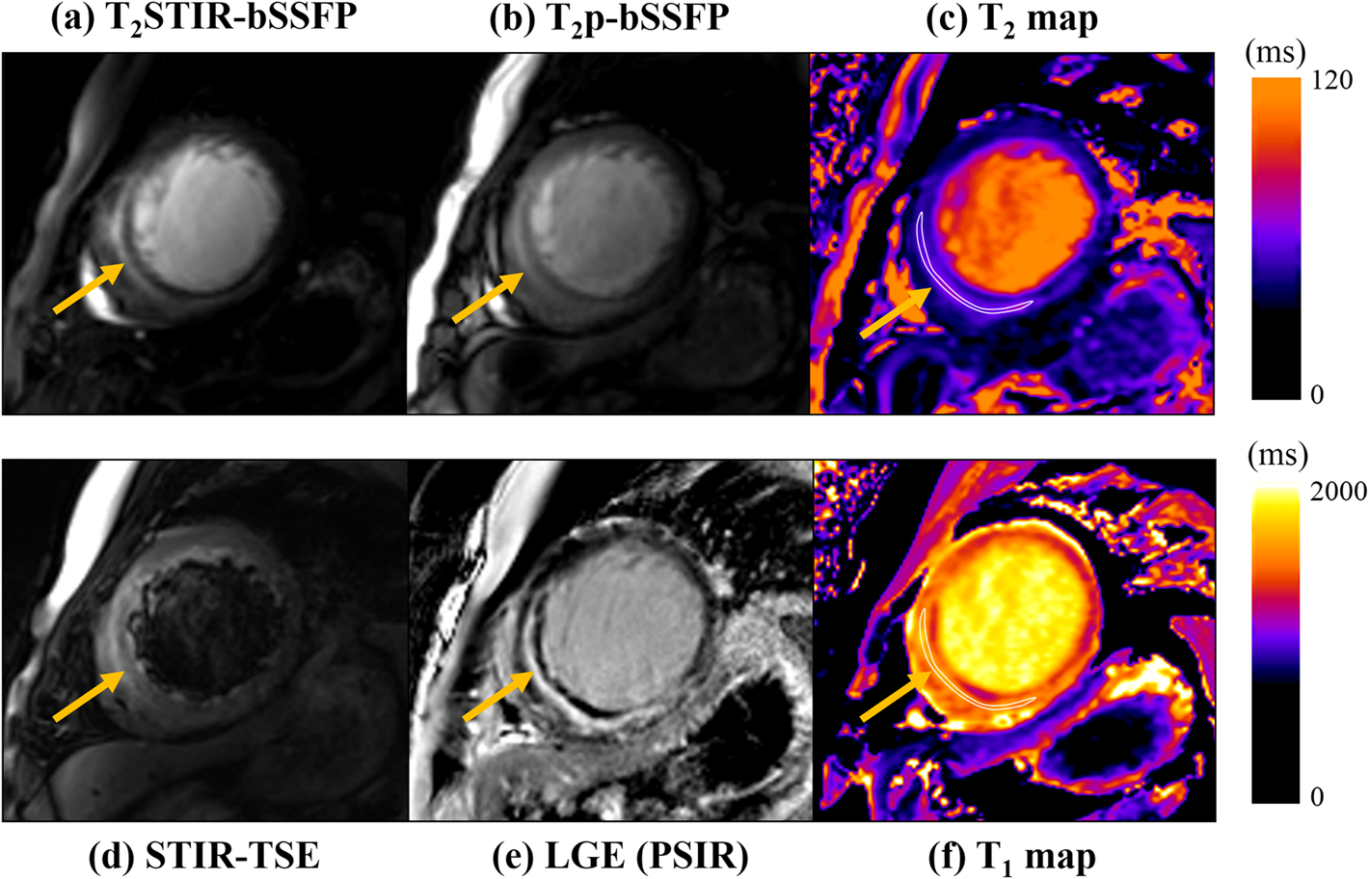
Images from another AMI patient. (**a**) T_2_STIR-bSSFP image (**b**) T_2_p-bSSFP image (**c**) The T_2_ map (**d**) The STIR-TSE image showed the edema surrounding a dark region that may correspond to microvascular obstruction. (**e**) The LGE image (**f**) The T_1_ map. The images of T_2_STIR-bSSFP and T_2_p-bSSFP were windowed to the same level

Figure 6 shows the images from another AMI patient. T_1_ and T_2_ elevation from the parametric maps (Fig. 6(c) and (f)) at the septal wall suggested the presence of edema. Enhancement in the LGE image (Fig. 6(e)) matched the position of edema in the parametric maps. Again, the signal for remote myocardium in the T_2_STIR-bSSFP image was lower than that in the T_2_p-bSSFP image, while the reverse was true for edema. The contrast improvement in T_2_STIR-bSSFP compared with T_2_p-bSSFP was obvious. The edematous region also appeared in STIR-TSE (Fig. 6(d)). However, the signal of the normal myocardium is highly non-uniform and might be affected by coil sensitivity variation. The T_2_STIR-bSSFP images from the remaining 5 patients are shown in Additional file 1: Figure S2.

## Discussion

This study proposes a novel magnetization preparation module, T_2_STIR, for single-shot myocardial edema imaging and demonstrates its relevance to myocardial edema imaging. Compared with the T_2_p module, the T_2_STIR module increases the contrast between edematous tissues and normal myocardium without increasing SAR. The simulation and phantom experimental results agreed well with each other. Both showed improved contrast of T_2_STIR-bSSFP compared with T_2_p-bSSFP and T_2_ mapping. In vivo experiments showed that T_2_STIR-bSSFP outperformed the other two common edema imaging methods: in healthy subjects, the technique showed an improved CNR and contrast ratio between blood and myocardium compared with T_2_p-bSSFP. In patients, T_2_STIR-bSSFP had higher CNR than T_2_p-bSSFP and the highest contrast ratio compared to T_2_p-bSSFP and T_2_ mapping.

A T_2_ preparation pulse combined with inversion recovery has been proposed in previous studies [32–36]. However, the inversion times (TI) in these applications were chosen to null tissues other than fat (normal myocardium, blood, etc.). The T_2_STIR module proposed here is novel in that (1) TI is selected to suppress the tissue with the shortest T_1_ (fat in this case) and (2) the inversion pulse is integrated with the T_2_ preparation module and reduces the SAR. Although blood is not nulled in T_2_STIR-bSSFP, no flow artifact was observed in the imaging experiments, especially in the long axis view. The reason is that the T_2_STIR pulse is nonselective. Additionally, image acquisition is performed in mid-diastole when blood flow is minimal. Flow artifacts are therefore much less likely.

A key feature of T_2_STIR-bSSFP compared with other gradient echo-based edema imaging sequences is its robust fat suppression. It removes the dark rim at the fat-water interface and improves tissue visualization (Fig. 5(a)). Fat suppression in T_2_STIR-bSSFP also helps differentiate pericardial fluid (common in AMI patients) from epicardial fat, both bright in bSSFP images (Fig. 5(b)). While fat suppression is possible with spectrally selective pulses in T_2_p-bSSFP, the technique is sensitive to main field inhomogeneity and increases SAR, especially at a high field. Theoretically, the performance of fat suppression in T_2_STIR-bSSFP would be affected by the T_1_ and T_2_ values of fat according to Eq. [2]. Incomplete inversion and the excitation pulses from the bSSFP readout before the k-space center would also affect fat suppression. Our experience with the single-shot T_2_STIR-bSSFP from the in vivo imaging experiments using TE_prep_ = 60 ms revealed that a TI_fat_ ranging from 110 ms to 130 ms can effectively suppress fat. The choice of TI_fat_ is therefore quite forgiving in practice.

The proposed T_2_STIR magnetization preparation module would be attractive at a main magnetic field beyond 3 T for two reasons. First, the T_2_STIR module will have improved contrast. As T_1_ increases with the magnetic field, the T_1_ difference between edematous and normal myocardium is increased. The contrast between the two tissues would therefore increase at a higher magnetic field with the T_2_STIR module. Additionally, the longer T_1_ of fat at higher field strength allows the use of longer TE_prep_ (while TI_fat_ satisfies Eq. [2]), further improving the edema contrast of this technique. Second, T_2_STIR-bSSFP is generally more favorable to other multishot edema imaging techniques in terms of SAR. For instance, single-shot T_2_STIR imaging is only 33% that of T_2_ mapping. However, the bSSFP readout is prone to banding artifact caused by field inhomogeneity, especially at 3 T and beyond. The banding artifact may be avoided or moved out of the ROI by using local shimming and frequency scout [37]. Alternatively, spoiled gradient readout can be used instead of bSSFP readout in single-shot T_2_STIR imaging if banding artifacts still persists.

While T_2_STIR-bSSFP shows the highest contrast ratio among the three edema imaging sequences compared and holds great promise for edema imaging in STEMI patients, it is not meant to replace T_2_ mapping. The latter method can identify global changes in T_2_ values, such as diffuse myocarditis and Takotsubo cardiomyopathy [38], for which T_2_STIR-bSSFP is not applicable. In fact, the two techniques may be used together: the single-shot T_2_STIR-bSSFP technique can identify edematous regions of the whole heart without requiring breath-holding. The T_2_ mapping technique can then be performed at the selected location where edema is found. Taking into account the time needed for full T_1_ recovery between consecutive slices, T_2_STIR-bSSFP can cover the whole heart (8 slices) in approximately 40 s with no need for breath-hold.

STIR-TSE images are susceptible to coil sensitivity variation. The same is true for T_2_p-bSSFP, in which the SI of edema in the images is only 25–50% [8] higher than that of normal myocardium. In T_2_STIR-bSSFP, the SI of edema is 60–220% higher than of normal myocardium in both phantom and patients without coil sensitivity correction. Coil sensitivity correction is therefore optional, although it helps to further improve image contrast. In T_2_ mapping, curve fitting removes the effect of coil sensitivity on T_2_ values, but respiratory motion or imperfect breath-holding may increase the variability of T_2_ values. Also, the estimated T_2_ value in T_2_ mapping may vary with arrhythmia, especially for long T_1_ tissues (Additional file 1: Table S1).

In this study, the performance of three bSSFP-based sequences was evaluated using the CNR and the contrast ratio. SNR measurement in magnitude images is not accurate due to a few reasons. The use of phased array coils results in spatially non-uniform signals in magnitude images but does not affect the parametric maps. In vivo noise measurement is not easy either. The use of parallel imaging results in spatially varying noise and makes noise measurement nontrivial [39]. Image noise also depends on imaging parameters such as voxel size and readout bandwidth. In this study, noise was measured as the SD of normal myocardium [31] and was applied to both qualitative images and parametric maps. Because it is difficult to accurately measure SNR in vivo, the contrast ratio is used to complement the CNR in quantifying image contrast. The contrast ratio is independent of noise and imaging parameters used. This metric has been shown elsewhere [40, 41] to be a better indicator than CNR in describing the ability of an imaging technique to differentiate two types of tissues. Additionally, the metric is applicable to both qualitative images and parametric maps.

In addition to the SNR measurement method, the study has other limitations. Though T_2_STIR-bSSFP images show edema and its positions correctly in all patient cases, its use for quantifying the extent of edema may need further study. Additional file 1 show that when the mean + 2SD was used empirically as the criterion for edema area quantification, errors exist in some cases. While the new technique has better image contrast than other bSSFP techniques compared, the spatial variation of coil sensitivity over the T_2_STIR-bSSFP images may affect accurate delineation of edematous areas to some extent. In addition, the low contrast between the edema region and the adjacent bright blood pool may make the detection of sub-endocardial edema using T_2_STIR-bSSFP difficult. A single-shot dark-blood T_2_STIR-bSSFP may be a good way to address this issue and will be investigated in our future work. Nevertheless, this study is a proof of concept, designed to demonstrate the feasibility and potential of T_2_STIR-bSSFP. It was not a clinical study. Our future work will evaluate the strength, limitations and the optimal threshold value for the segmentation of edema in T_2_STIR-bSSFP images, and establish the clinical relevance of T_2_STIR-bSSFP in a clinical setting through a large patient cohort.

## Conclusion

This study proposes T_2_STIR-bSSFP that exploits the elevation of both T_1_ and T_2_ values of edema to increase the contrast between edematous and normal myocardium. The single-shot technique provides a fast and robust method for myocardial edema imaging with improved contrast compared with several other edema imaging techniques at 3 T.

## Supplementary information

**Additional file 1.** 1. The formulas for Signal difference, SNR, CNR and contrast ratio. 2. The extent of edema identified using different methods. 3. The effect of arrhythmia on T_2_ maps.

## Data Availability

The datasets are available from the corresponding author upon reasonable request.

## Abbreviations

AFP: Adiabatic full passage
AHP: Adiabatic half passage
AMI: Acute myocardial infarction
bSSFP: Balanced steady state free precession
CMR: Cardiovascular magnetic resonance
CNR: Contrast-to-noise ratio
ECG: Electrocardiogram
IRB: Institutional review board
LFA: Linear flip angle pulses
LGE: Late gadolinium enhancement
mBIR-4: Modified B_1_-insensitive rotation pulse
rAHP: Reverse adiabatic half passage
ROI: Region of interest
SAR: Specific absorption rate
SD: Standard derivation
SI: Signal intensity
SNR: Signal-to-noise ratio
SPAIR: Spectral attenuated inversion recovery
STEMI: ST elevation myocardial infarction
STIR: Short tau inversion recovery
T_2_p-bSSFP: T_2_-prepared balanced steady state free precession
T_2_STIR-bSSFP: T_2_ prepared inversion with short tau inversion recovery balanced steady state free precession
TSE: Turbo spin echo
