# Supplementary material for: T2STIR preparation for single-shot cardiovascular magnetic resonance myocardial edema imaging
Source: J Cardiovasc Magn Reson. 2019 Nov 21;21:72. doi: 10.1186/s12968-019-0583-y (PMC6873416; doi:10.1186/s12968-019-0583-y)
Supplement: Supplementary file 1 — Additional file 1. 1. The formulas for Signal difference, SNR, CNR and contrast ratio. 2. The extent of edema identified using different methods. 3. The effect of arrhythmia on T2 maps. [file 12968_2019_583_MOESM1_ESM.docx]

**Supplemental Materials**

1. **Signal difference, SNR, CNR and contrast ratio**

The signal difference, SNR, CNR, and contrast ratio for T_2_p-bSSFP and T_2_STIR-bSSFP were calculated as follows:

Signal difference = SI of edema – SI of normal myocardium (S1)

SNR = SI/SD of normal myocardium (S2)

CNR = Signal difference/SD of normal myocardium (S3)

Contrast ratio = SI of edema/SI of normal myocardium (S4)

Following Duan C, et.al. (Magn Reson Med 2018, DOI:10.1002/mrm.27636 ) and ref [31], the SNR, CNR and contrast ratio for the T2 maps were defined as follows:

SNR_T2_ = T_2_ value/SD of normal myocardial T_2_ (S5)

CNR_T2_ = $\frac{\text{T}\text{2}\text{ of edema – T}\text{2}\text{ of normal myocardium}}{\text{SD of normal myocardial T}\text{2}}$ (S6)

Contrast ratio_T2_ = T_2_ of edema/T_2_ of normal myocardium (S7)

Note that the SNR definition is also the inversion of coefficient of variation, which shows the variability of the measurements.

1. **Extent of edema**

We calculated the areas of the edematous regions using the so-called “2SD method” (Ferreira VM et al., JACC Cardiovasc Imaging 6(10):1048, 2013). In this method, the LGE images were used to identify remote myocardium. The mean and standard deviation (SD) of normal myocardium in the T2 map, T2prep-bSSFP and T2STIR-bSSFP were then found. The area with values above mean + 2SD of the remote myocardium was classified as edema. The extent of edema was defined by the ratio of edema size and left ventricular mass (%LVM).

The agreement of the edema extents obtained from T_2_STIR-bSSFP images, T_2_p-bSSFP images, and T_2_ maps were compared by Bland-Altman analysis. To assess the reproducibility measurements, the edematous areas measured from images acquired using different techniques were reviewed by a second observer (3-year experience in CMR). The intra-class correlation coefficient (ICC) analysis with absolute agreement was used to assess inter-observer agreement for edema extent.

The Bland-Altman plots of edema extent between different techniques are shown in Supporting Figure S1. Given the small sample size, the edema extents of T_2_STIR-bSSFP were in reasonable agreement with that of T_2_ map (bias: 2.3%, 95% Confidence Interval (CI): -38.1%~42.6%) but higher than that of T_2_p-bSSFP (bias: 5.1%, CI: -42.2%~32.0%). Since the total sample size is small (n = 7), the one outlier in the dataset greatly enlarge the confidence interval (CI) in Bland Altman plots. Excluding the outlier, the edema extents of T_2_STIR-bSSFP show good agreement with that of T_2_ map (bias: -3.2%, 95% Confidence Interval (CI): -8.7%~2.4%).

The extents of edema identified by the two observers using both sets of images showed strong agreement (ICC: 0.91, CI: 0.80-0.96) in terms of inter-observer agreement.


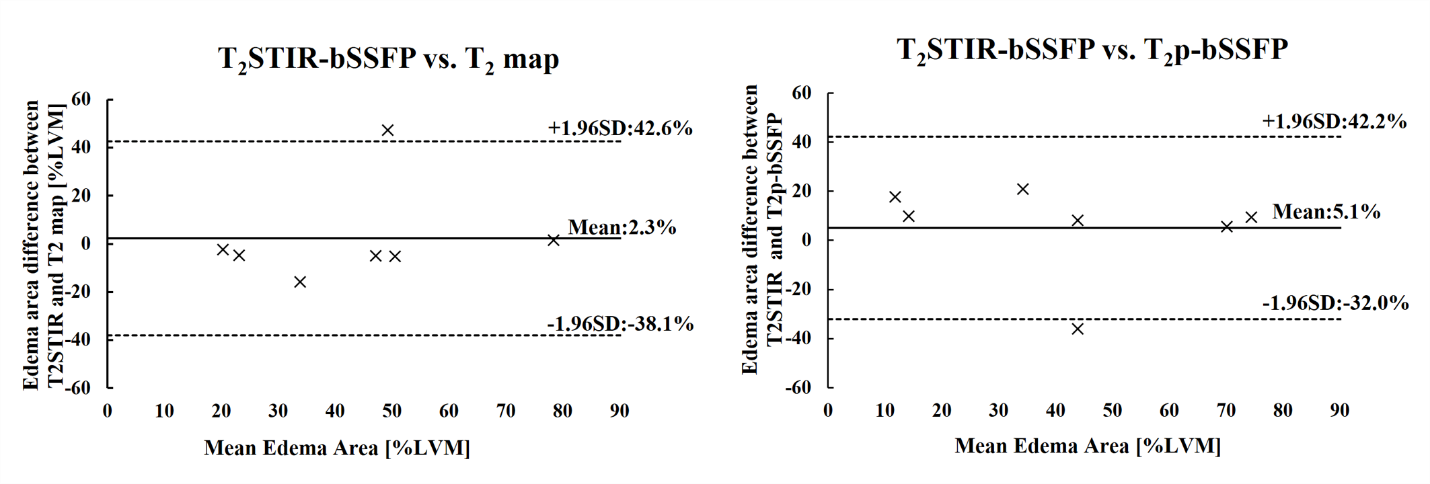


Figure S1. The Bland-Altman plots of edema extents obtained from images of different techniques.


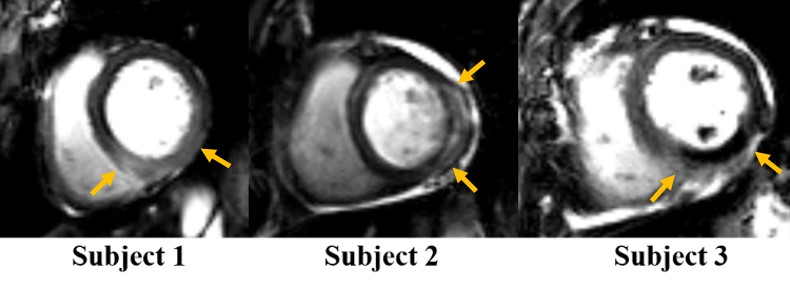


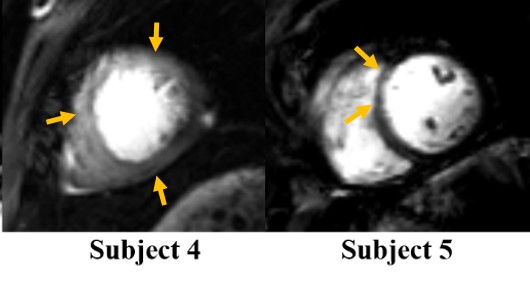


Figure S2.The T_2_STIR-bSSFP images from the remaining 5 patients collected in the study. The yellow arrows indicate the edema areas.

1. **Effect of arrhythmia on T_2_ maps**

The effect of arrhythmia on T_2_ values obtained by T_2_ mapping at 3T was simulated. In the simulation, the T_1_ and T_2_ values used were the same as in the simulation of the manuscript, i.e., 1139 ms and 52 ms for normal myocardium and 1434 ms and 75 ms for edematous tissue, respectively. The imaging parameters were: bSSFP readout with centric k-space ordering, 10 linear flip angle (LFA) pulses for catalysis, TE/TR = 1.3/2.6 ms, flip angle = 35°, TE_prep_ = 0, 25, and 55ms, 3RR interval between two consecutive acquisitions. We tried different heart rate conditions and their effects on T_2_ values of normal myocardium and edema.

Table S1 shows the estimated T_2_ values using different RR intervals. The T_2_ values in normal myocardium do not vary too much when the RR interval is regular. However, the T_2_ value of edema decreases by more than 10% in arrhythmia. The main reason for this is the longer T_1_ in edema compared to normal myocardium. When arrhythmia leads to a shortened RR interval, long T_1_ tissue, e.g. edema, would have reduced recovery time. Magnetization for the subsequent acquisition cycle will be reduced. This leads to an underestimation of T_2_ values in edema. The T_2_ value of edematous tissue therefore varies with arrhythmia while T_2_ values of normal myocardium may only be minimally affected.

| Heart rate pattern | Measured T_2_ values | | Reference T_2_ values | |
| --- | --- | --- | --- | --- |
|  | Normal myocardium | Edema | Normal myocardium | Edema |
| “Regular” rhythm (RR=800ms) | 51ms | 72ms | 52ms | 75ms |
| Tachycardia (RR=500ms) | 49ms | 66ms |  |  |
| Bradycardia (RR=1300) | 53ms | 75ms |  |  |
| Arrhythmia  (regular RR=800ms, 1st interval =3RR, 2nd interval =2RR, i.e., skipped 1 heart beat) | 49ms | 67ms |  |  |

**Table S1**. The estimated T_2_ values for normal myocardium and edema by simulation using different RR intervals at 3T.
